# Pleiotropic effects of the twin-arginine translocation system on biofilm formation, colonization, and virulence in *Vibrio cholerae*

**DOI:** 10.1186/1471-2180-9-114

**Published:** 2009-05-31

**Authors:** Lijuan Zhang, Zhaoqin Zhu, Huaiqi Jing, Jingyun Zhang, Yanwen Xiong, Meiying Yan, Shouyi Gao, Long-Fei Wu, Jianguo Xu, Biao Kan

**Affiliations:** 1 State Key Laboratory for Infectious Disease Prevention and Control, Department of Diarrheal Diseases, National Institute for Communicable Disease Control and Prevention, Chinese Center for Disease Control and Prevention. Beijing 102206, PR China; 2 Laboratoire de Chimie Bacterienne, UPR9043, IBSM, CNRS, F-13402 Marseille Cedex 20, France

## Abstract

**Background:**

The Twin-arginine translocation (Tat) system serves to translocate folded proteins, including periplasmic enzymes that bind redox cofactors in bacteria. The Tat system is also a determinant of virulence in some pathogenic bacteria, related to pleiotropic effects including growth, motility, and the secretion of some virulent factors. The contribution of the Tat pathway to *Vibrio cholerae *has not been explored. Here we investigated the functionality of the Tat system in *V. cholerae*, the etiologic agent of cholera.

**Results:**

In *V. cholerae*, the *tatABC *genes function in the translocation of TMAO reductase. Deletion of the *tatABC *genes led to a significant decrease in biofilm formation, the ability to attach to HT-29 cells, and the ability to colonize suckling mouse intestines. In addition, we observed a reduction in the output of cholera toxin, which may be due to the decreased transcription level of the toxin gene in *tatABC *mutants, suggesting an indirect effect of the mutation on toxin production. No obvious differences in flagellum biosynthesis and motility were found between the *tatABC *mutant and the parental strain, showing a variable effect of Tat in different bacteria.

**Conclusion:**

The Tat system contributes to the survival of *V. cholerae *in the environment and *in vivo*, and it may be associated with its virulence.

## Background

In bacteria, transmembrane translocation, required for many newly synthesized proteins, can proceed through a number of routes depending on the nature of both the targeting signals and the folding state of substrates. In general, folded proteins are exported through the twin-arginine translocation (Tat) system [1]. Precursor proteins are directed to the Tat pathway by signal peptides that bear a characteristic consensus sequence, an unusually long S/T-R-R-x-F-L-K "twin-arginine" motif [2,3]. The most extensively characterized substrates for this pathway are trimethylamine N-oxide (TMAO) reductase, a soluble periplasmic enzyme, and dimethyl sulfoxide (DMSO) reductase, a membrane-bound multisubunit enzyme, which have twin arginine signal sequences [1].

The Tat pathway is structurally and functionally related to the pH-dependent protein import pathway of the plant chloroplast thylakoid membrane [2,4]. The Tat system of *E. coli *seems to operate with a similar mechanism as the Tat machinery of chloroplast thylakoids, as genes encoding HCF106 homologues are found in the complete genome sequences of some prokaryotes. Both pathways require three functionally distinct membrane-bound components, MttA, MttB, and MttC for HCF106, and TatA, TatB, and TatC for *E. coli *[5,6]. It is believed that TatB and TatC form a complex and are required for the recognition and binding of the twin-arginine signal peptide [7,8]. TatA is a homo-oligomer complex, which is recruited by the TatB-TatC complex and probably fulfills a channel function in the protein export process [9,10]. TatE, a TatA paralogue, functionally overlaps with TatA in *E. coli *[1].

The Tat pathway is the major pathway required for the translocation of cofactor-containing enzymes participating in the respiratory and photosynthetic electron transport chains [4]. Indeed, the Tat system may be a determinant of virulence in some bacteria, as deletion of the Tat system may lead to pleiotropic defects, including growth, motility, and the secretion of some virulent factors in pathogenic bacteria. For example, the system is important for the virulence of pathogens including *Pseudomonas aeruginosa *[11,12], *Agrobacterium tumefaciens *[13], *E. coli *O157:H7 [14], *Yersinia pseudotuberculosis *[15], and *Legionella pneumophila *[16,17]. However, the contribution of the Tat pathway to the survival and virulence of *Vibrio cholerae *has not been reported.

*V. cholerae *is the causative agent of the diarrheal disease cholera. To date, there have been seven recorded pandemics of this severely dehydrating diarrheal disease. The ability of *V. cholerae *to survive the passage through the human gastric acid barrier, to colonize the human intestine with its pili and other outer membrane proteins and polysaccharides, and to secrete the cholera toxin (CT) are all crucial components of the bacterial life cycle [18]. Secretion of proteins is critical for the pathogenicity of the organism and for its survival in the natural environment. The genome of *V. cholerae *El Tor contains the *tatABC *operon in chromosome I and the *tatA2 *(*tatE*) gene in chromosome II [19]. To analyze the function and the involvement of the Tat system in the survival and virulence of *V. cholerae*, we constructed chromosomal in-frame deletion mutations in *tatABC *and *tatE*. Our findings demonstrate that the *V. cholerae tatABC *genes function in the translocation of TMAO reductase. Moreover, we found that the mutation affected biofilm formation, attachment to HT-29 cells, and colonization of suckling mouse intestines. The flagellum biosynthesis and motility, outer membrane integrity, and growth rate in normal cultures of Tat mutants were not affected. We also observed that the mutation impaired the transcription of the toxin gene, as well as CT production, although the ratio of secreted toxin to toxin stored in the cytoplasm was the same in the mutant and in the wild type strain. Overall, the Tat system is associated with the survival, as well as the virulence of *V. cholerae*.

## Methods

### Bacterial strains, media, and growth conditions

The bacterial strains and plasmids used in this study are listed in Table 1. The *tatABC *deletion mutant N169-dtatABC strain was derived from the wild type O1 El Tor strain N16961 (Table 1). Both *E. coli *and *V. cholerae *cells were routinely grown at 37°C in Luria-Bertani broth (LB). For plate culture, LB was used with 1.5% agar (LBA). For the detection of CT production, *V. cholerae *were first grown under AKI conditions with sodium bicarbonate (1.5% Bacto Peptone, 0.4% yeast extract, 0.5% NaCl) at 37°C for 4 h, and the culture was then incubated overnight while shaking at 37°C [20]. Antibiotics were used at the following concentrations: ampicillin, 100 μg/ml; streptomycin, 100 μg/ml; and chloramphenicol, 30 μg/ml. The growth kinetics of the bacterial culture was measured spectrophotometrically with the optical density (OD) of the culture at 600 nm. Complementarity of the *E. coli tat *mutants complemented by the *V. cholerae tat *genes was analyzed by anaerobic growth in M9-TMAO minimal media. The components of the M9-TMAO medium (for a final volume of 1 liter) in this study are listed below: 12.8 g Na_2_HPO_4_; 3.0 g KH_2_PO_4_; 0.5 g NaCl; 1.0 g NH_4_Cl; 2 ml 1 M MgSO_4_; 0.1 ml 1 M CaCl_2_; 1 ml 1% thiamine; 1 ml molybdenum-selenium solution (1 mM potassium selenite and 1 mM ammonium molybdate); 0.05 ml Glycerol; 0.04 g TMAO. Strains were cultured at 37°C without shaking. The OD_600 _values were taken 22 hours after inoculation.

**Table 1:** Bacterial strains and plasmids used in this study

Strains or plasmids	Relevant genotype and/or phenotype	Source or reference
*V. cholerae*		
N16961	Serogroup O1, El Tor biotype	Our lab store
N169-dtatABC	*tatABC *deletion mutant from N16961	This study
N16961(pBAD24)	N16961 transformed with vector pBAD24	This study
N169-dtatABC(pBAD24)	N169-dtatABC transformed with pBAD24	This study
N169-dtatABC-cp	N169-dtatABC complemented with pBAD-TatABC	This study
N169-dtatABC-BCcp	N169-dtatABC complemented with pBAD-TatBC	This study
N169-dtatE	*tatE *deletion mutant from N16961	This study
N169-dtatABCE	*tatABC *and *tatE *double deletion mutant from N16961	This study
N169-dtatABCE-BCcp	N169-dtatABCE complemented with pBAD24 carrying *tatBC*	This study
N169-dtatB	*tatB *deletion mutant from N16961	This study
N169-dtatC	*tatC *deletion mutant from N16961	This study
		
*E. coli*		
SM10 λ*pir*	*thi thr leu tonA lacY supE recA*::RP4-2-Tc::Mu Km	21
JARV16A (dtatAE)	*tatA *and *tatE *double deletion mutant from JARV16A	34
MCMTAA(dtatB)	*tatB::Kan *mutant from MCMTAA	34
B1LK0A (dtatC)	*tatC *deletion mutant from B1LK0A	34
DADEA (dtatABCDE)	*tatABCD *and *tatE *double deletion mutant from DADEA	34
		
Plasmids		
pCVD442	Suicide vector, *ori *R6K, Amp^r^, *sacB*	21
pDS132	Suicide vector, *ori *R6K, from pCVD442, Cm^r^, *sacB*	22
pT1	714 bp *EcoR*I-*Kpn*I fragment A of *tatA *cloned into pUC18	This study
pT2	461 bp *Xba*I-*Pst*I fragment B of *tatC *cloned into pT1	This study
pT3	801 bp fragment of *cat *cloned into *Sma*I site of pT2	This study
pCT4	1,976 bp fragment of 'A-*cat*-B' cloned into *Sph*I site of pCVD442	This study
pUC18C	intact *tatABC *and upstream fragment cloned between *EcoR*I and *Sac*I site of pUC18	This study
pBAD24	pMB1-derived plasmid, Amp^r^, *araBAD*	23
pTatABC-301	intact *tatABC *fragment of *E. coli *cloned into pBAD24	This study
pBAD-TatABC	intact *tatABC *fragment of N16961 cloned into pBAD24	This study
pBAD-TatBC	*tatBC *fragment of N16961 cloned into pBAD24	This study
pBAD-TatE	*tatE *fragment of N16961 cloned into pBAD24	This study

### Construction of the tat deletion mutants of *V. cholerae *N16961 by allelic replacement

To inactivate the *tatABC *genes of strain N16961, fragment A, which contains the 5' portion of gene *tatA *and its upstream region, was amplified and digested with the enzymes *Eco*RI and *Kpn*I and ligated between the *Eco*RI and *Kpn*I sites of the pUC18 vector, generating the plasmid pT1 (Table 1). The 461 bp fragment B, which includes the 3' portion of gene *tatC *and its downstream region, was amplified and ligated between the *Xba*I and *Pst*I sites of the vector pT1, generating the plasmid pT2 (Table 1).

The chloramphenicol gene (*cat*) was amplified and ligated into the *Sma*I site of pT2, generating the plasmid pT3. To create a deletion-insertion allele, pT3 was digested with the enzyme *Sph*I, and a 1976 bp fragment of "A-*cat*-B" was gel purified and subsequently ligated into the *Sph*I site of pCVD442 [21]. The resulting recombinant plasmid, pCT4, was then transferred by conjugation from *E. coli *SM10 *λpir *[21] into the *V. cholerae *strain N16961. Mutant strains were selected on chloramphenicol plates with sucrose but without NaCl at 30°C, by SacB counter-selection strategy. The mutant strain, N169-dtatABC, which contains a mutation in *tatABC*, was confirmed by PCR and sequencing. The intact sequences of the neighboring genes in the upstream and downstream regions of *tatABC *were also confirmed.

To complement the *tatABC *deletion, a DNA fragment containing the *tatABC *gene and a 206 bp upstream fragment was amplified. The resulting fragment was then ligated into the *Eco*RI/*Sac*I digested vector, pBAD24. After transformation of the recombinant plasmid into N169-dtatABC cells, the complemented strain N169-dtatABC-cp was obtained.

To test the functions of different genes of the Tat system, we constructed four more chromosomal in-frame deletion mutants (N169-dtatB, N169-dtatC, N169-dtatE and N169-dtatABCE, see Table 1) by allelic replacement and SacB counter-selection strategy with the suicide plasmid pDS132 [22], and two other complemented strains (N169-dtatABC-BCcp and N169-dtatABCE-BCcp, see Table 1) with the expression plasmid pBAD24 [23], according to the strategies used above (in deletion mutation through allelic replacement with pDS132, the marker of *cat *gene was not used any more). The primers used to construct the mutants and complementary strains were listed in the Additional file 1. Reverse transcription-PCR were used to detect the gene transcription in these mutants and complement strains in LB culture.

### Enzymatic assay

The test for trimethylamine-N-oxide (TMAO) reductase activity is based on the oxidation of reduced methyl viologen, coupled to the reduction of TMAO to trimethylamine [24,25]. To analyze the cellular distribution of TMAO reductase, periplasm and spheroplasts were prepared by the lysozyme-EDTA-cold osmoshock method [25]. The prepared fractions of periplasm and cytoplasm were confirmed by using western blotting, with the antibodies to β-lactamase and GroEL (Abcam). Strain N16961 was transformed with plasmid pBAD24 to express β-lactamase and obtain ampicillin resistance. IRDye 800CW goat anti-mouse IgG (LI-COR Bioscience) was used as the second antibody. The bands were scanned with the Odyssey Infrared Imaging Systems (LI-COR Bioscience). The mixture was then resolved ret by 12% non-denaturing polyacrylamide gel (polyacrylamide gel without denaturant SDS) electrophoresis, and TMAO reductase activity was subsequently visualized on non-denaturing polyacrylamide gels. For this purpose, the gels were placed in a nitrogen atmosphere in a plate containing 25 ml of potassium phosphate buffer (100 mM, pH 6.5), 0.5 ml of 0.22 g/ml methyl viologen solution, and a small amount of Na_2_S_2_O_4 _dissolved in 0.01 M NaOH. The gels, uniformly colored blue by reduced methyl viologen, were then incubated in 25 ml of phosphate buffer (100 mM, pH 6.5) supplemented with 0.5 ml of 0.25 g/ml TMAO solution. The resulting clear bands on the blue background indicate the presence of active TMAO reductase in the gel.

### Growth assessment of strains in M9-TMAO media

The overnight cultures of different *tat *genes deletion mutants and complement strains (listed in Table 1) and the wild type strain N16961 were diluted 1:100 and incubated in fresh LB to OD_600 _more than 0.8. Then the culture of each was adjusted with LB to OD600 of 0.8. Then they were then diluted 1:100, and 50 μl of each culture was transferred into M9-TMAO media and subsequently cultured statically at 37°C in the anaerobic jar (Oxoid). The vacuum extractor was used to extract the air in the anaerobic jar to lower atmospheric pressure (-10 millimeters of mercury), and then H_2 _and CO_2 _were inflated to normal atmospheric pressure. The culture was grown for 24 h, and then the OD_600 _of each culture was determined.

### Motility assay

Motility of N16961 and N169-dtatABC cells was tested on 0.3% minimal motility agar containing 1% peptone and 0.5% NaCl (wt/v). Briefly, cell cultures grown in LB broth overnight at 37°C were diluted 1:1000. Cell cultures were then grown to OD_600 _0.2. Subsequently, each strain was inoculated onto the surface of the motility U type tubes. Motility was examined after 12 h and 16 h of incubation at 37°C. The percentage of the length of growth diffusion in the agar of the mutant strain N169-dtatABC compared to the wild type strain was calculated. At least five independent motility assays were carried out for each strain and condition.

### Outer membrane integrity assay

We detected the outer membrane integrity according to the method of reference [26]. The wild type strain N16961 and the Tat mutant strain N169-dtatABC were cultured overnight and then diluted 1:100 into fresh LB and grown to OD_600 _0.4. Five milliliters of fresh LB was added into each tube, and SDS or Gentamicin (Get) was added to final concentrations of 0 to 2.5% and 0 to 500 μg/ml, respectively. Experiments were performed in triplicate for N16961 and Get. After SDS or Get addition, all tubes were grown at 37°C for 3 h at 250 rpm, after which the OD_600 _of each culture tube was measured. We defined the OD_600 _of the wild type strain cultured in LB without SDS and Get as 100%. The OD_600 _values of the other conditions were converted to the percentage of OD_600 _of the wild type strain cultured in LB without SDS and Get. To determine whether the outer membrane of the mutants was destroyed, the results are plotted as SDS or Get dilution on the X-axis and OD_600 _percentage on the Y-axis [26].

### Flagellum extraction and quantification

Bacterial cells were recovered from a 600 ml LB culture of N16961 and N169-dtatABC incubated overnight at 37°C and then centrifuged for 5 min at 10,000 g. The pellets were resuspended in PBS buffer and vortexed for 5 min, with a 30 s interval after 2.5 min. The supernatants were centrifuged again for 10 min at 15,000 g. After recovery of the supernatants, SDS was added (0.1% wt/v). The flagellum pellets were obtained by centrifugation at 100,000 g for 2 h at 4°C. The supernatants were removed, and the flagellum filaments were resuspended in 50 μl of HEPES buffer (10 mM HEPES, 10 μM EDTA pH 8.0, 200 μM CaCl_2_). Before the flagella were detached from the N16961 and N169-dtatABC cells, we calculated the wet weight of each cell type. To quantify the extracted flagellum proteins, the flagellum extracts from N16961 and N169-dtatABC cells were equated by the wet weight of the collected cells. The concentration of the flagellum extraction was quantified with the BSA standard curve by Bradford assay. Purity of the flagellum preparations was assessed by denaturing SDS-PAGE. Flagellum extraction and quantification were performed in triplicate.

### Biofilm formation assay

In a quantitative biofilm formation assay, both primary attachment and accumulation in multilayered cell clusters, which together lead to biofilm formation, can be measured by altering the incubation time of the bacteria. Biofilm assays were done according to the protocol of Loo *et al. *[27] with minor modifications. Briefly, overnight cultures of N16961 and dtat-N169 cells were diluted 1:100 into fresh LB medium and grown at 37°C to OD_600 _0.5, both under aerobic and anaerobic conditions. The cultures were then again diluted 1:100 into fresh LB, and 200 μl of the cell suspension was placed into separate wells of a 96-well (flat bottom) cell culture plate (Costar 3595, Corning). Wells containing fresh growth medium were used as negative controls. Plates were incubated at 37°C under both aerobic and anaerobic conditions for 6 to 72 h. The artificial anaerobic condition was generated by an anaerobic jar (Oxoid) where the plates were incubated. The vacuum extractor was used to extract the air in the anaerobic jar to lower atmospheric pressure (-10 millimeters of mercury), and then H_2 _and CO_2 _were inflated to normal atmospheric pressure. Before biofilm quantification, growth was assessed by measuring the absorbance of cultures in the wells at 595 nm using GENios (TECAN). For this purpose, media and unattached bacterial cells were decanted from the wells after 5 min of agitation, and the remaining planktonic or loosely bound cells were removed by gentle rinsing with 200 μl of sterile distilled water. The plates were then blotted on paper towels and air-dried. The adhering bacteria were stained with 225 μl of 0.1% crystal violet for 15 min at room temperature. After two rinses, each with 250 μl of water, the bound dye was extracted from the stained cells using 250 μl of 99% ethanol. The plates were then agitated for 15 min to fully release the dye. Biofilm formation was quantified by measuring the absorbance of the rinsed solution at 595 nm with GENios. The data were obtained in triplicate tests, and seven wells were measured for each strain (N16961 and N169-dtatABC) and in each test.

### Cell culture and bacterial attachment assay

HT-29 (human colon adenocarcinoma) cells were grown in Dulbecco modified Eagle medium supplemented with equal volumes of F-12 nutrient mixture (Ham) powder (DMEM/F12 culture), 5% fetal bovine serum, and 1% Pen-Strep (GibcoBRL) under 5% CO_2 _[28]. The cultures of N16961 and N169-dtatABC cells were adjusted to the same optical density at 600 nm (1.0). A confluent HT-29 cell monolayer was infected with the bacterial mixture (1 mL LB containing 10^6 ^CFU of N16961 and 10^6 ^CFU N169-dtatABC) and incubated at 37°C. For quantification of the attached bacteria, a 6-well cell culture plate was used, the monolayers and attached bacteria were washed three times with PBS and incubated for 30 min in a 1% Triton X-100 solution. The resulting bacterial suspensions were appropriately diluted with LB and plated onto plates containing thiosulfate citrate bile salts sucrose (TCBS) agar and TCBS agar supplied with 15 μg/ml chloramphenicol. The competitive attachment ratio was calculated according to the following formula (the ratios were from 6 wells of repeat):

Competitive attachment ratio = (average number of colonies on TCBS plates – average number of colonies on chloramphenicol plates)/average number of colonies on chloramphenicol TCBS plates.

For the immunofluorescence assay, glass slides were placed in each well of a six-well plate (Corning) before the wells were inoculated with HT-29 cells. An HT-29 confluent monolayer was infected with 1 ml of N16961, N169-dtatABC, or N169-dtatABC-cp (10^6 ^CFU each) and incubated at 37°C for 4 h. The monolayers and attached bacteria were washed three times with PBS. Cells were then fixed using 2% polyformaldehyde. The monoclonal antibodies against the *V. cholerae *serogroup O1 were added into cells. The plates were incubated at 37°C for 1 h and washed three times with PBS. FITC-labeled IgG1 (1:1500 dilution in PBS) was added to each well. The plates were incubated at 37°C for 30 min and then inspected with the confocal microscope (LSM510META, Zeiss).

### Suckling mouse intestinal colonization assay

Suckling mouse intestines were infected with *V. cholerae *as described by Baselski and Parker [29] with slight modifications. Briefly, the overnight cultures of N16961 and N169-dtatABC cells were diluted in LB to an equal OD_600_. Five- to 7-day-old suckling Balb/C mice (separated from their mothers) were intragastrically inoculated with 100 μl of N16961 and N169-dtatABC cultures. The bacterial titers of each inoculum were determined by plating serial dilutions of the inocula. Infected mice were kept at 24°C in the absence of their mothers. Mice were sacrificed 16 h after inoculation. Whole intestines were removed, cut into short segments, and then mechanically homogenized in 4.5 ml of LB containing 20% (v/v) glycerol. Serial dilutions were plated onto TCBS agar (to isolate N16961 and N169-dtatABC) and TCBS agar supplemented with 50 μg/ml chloramphenicol (to isolate N169-dtatABC) to count the *V. cholerae *CFU per dilution. The attachment competitive ratio was calculated according to the same formulation as that of the HT-29 cell attachment model. Twelve suckling mice were used for the repeat of attachment assay.

### Assay of CT production by GM1-ELISA

CT production in culture supernatants was estimated in *V. cholerae *strains (N16961, N169-dtatABC, and N169-dtatABC-cp) incubated with AKI media (containing 1.5% Bacto peptone, 0.4% yeast extract-Difco, 0.5% NaCl and 0.3% NaHCO_3_), cultured at 37°C for 4 h in a stationary test tube and then for 16 h in a shaken flask, and measured by GM1-ELISA [30]. In our study, the medium used for all cultures was AKI with 0.3% sodium bicarbonate. To determine CT production, the strains incubated under static conditions for 4 h at 37°C were poured into flasks with a 20-fold greater volume than the tubes to continue growth at 37°C for 18 h with shaking at 220 rpm. All culture supernatants were concentrated 10-fold with PEG6000. A standard curve was generated using the purified B subunit of CT. As a second antibody, the monoclonal antibody against the B subunit of CT was added. Color intensity was measured at 492 nm in an ELISA reader (Bio-Rad). Three independent triplicate repeats were done for each strain.

### Transcription analysis of the *ctxB *gene in N16961 and N169-dtatABC cells

The overnight cultures of N16961 and N169-dtatABC cells were re-cultured to OD_600 _1.0 with fresh LB, and then 1:100 dilutions were transferred into AKI medium. The medium used for all cultures was AKI complemented with 0.3% sodium bicarbonate. To determine the *ctxB *transcription levels, the strains incubated under still conditions for 4 h at 37°C were poured into flasks with a 20-fold greater volume than the tubes to continue growth at 37°C for 18 h with shaking at 220 rpm. The RNeasy Mini Kit (Qiagen) was used to extract total RNA from 1 ml of bacterial cultures. The RNase-free DNase set Kit (Qiagen) was used to eliminate DNA. RNA samples were diluted to 1 ng/μl in order to obtain the template for RT-PCR after quantification. Primers 5'-CGC ATG AGG CGT TTT ATT ATT C-3' and 5'-AAA GCG ATT GAA AGG ATG AAG G-3' were used to amplify *ctxB *gene. The housekeeping gene *thyA *(primers 5'-ACA TGG GAC GCG TGT ATG G-3' and 5'-ATA TGA CCA CCA TCA GGC TTA GC-3') and 16S-rDNA (primers 5'-TTG ACA TCC AGA GAA TCT AGC GG-3' and 5'-TTA ACC CAA CAT TTC ACA ACA CGA-3') were selected as the references. RNA extraction and RT-PCR quantification were done in triplicate for each strain. 2^-ΔΔCt ^method was used to calculate the Ct difference of *ctxB *between N16961 and N169-dtatABC, with the existence of the control genes. ΔΔCt = (Ct_N169-dtatABC(*ctxB*) _- Ct_N169-dtatABC(reference)_) - (Ct_N16961 (*ctxB*) _- Ct_N16961(reference)_), in it Ct_N169-dtatABC(reference) _and Ct_N16961 (reference) _mean the average of Ct values of *thyA *or 16s-rDNA gene of the N169-dtatABC and N16961 samples respectively, Ct_N169-dtatABC(*ctxB*) _and Ct_N16961(*ctxB*) _mean the average of Ct values of *ctxB *gene of the N169-dtatABC and N16961 samples respectively. 2^-ΔΔCt ^means the times of *ctxB *transcription of N169-dtatABC compared to N16961.

## Results

### *V. cholerae *has a functional Tat system

**Figure 1:**
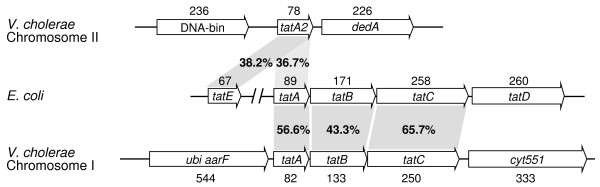
**Sketch of the chromosomal regions encoding *tat *genes in *E. coli *and *V. cholerae***. This sketch compares the structure of the *tat *gene clusters and the amino acid sequences between the *V. cholerae *El Tor strain N16961 and *E. coli*. The numbers near the arrowheads of the ORFs signify the length in amino acids, and the percentages indicate the amino acid identity of the compared genes connected with grey squares.

The genetic structure and composition of the *tat *genes vary in different bacteria [31]. We analyzed the genome sequence of *V. cholerae *N16961 and found the genes *tatA*, *tatB*, and *tatC *in chromosome I, and *tatA2 *in chromosome II (VC0086 and VCA0533 were annotated as *tatA *and *tatA2*, respectively). These genes encode four proteins with a high degree of homology to the *E*. *coli *K-12 *tat *genes, ranging from 43.3 to 65.7% amino acid identity (Fig. 1). In addition to the *tat *genes, the cytochrome c551 peroxidase gene (VC0089) was found in the downstream region of the *tatABC *operon, and the ubiquinone biosynthesis protein Aarf gene (VC0085) was found in the upstream region of the *tatABC *operon. No homologue of the previously designated *tatD *of *E. coli *was detected in the *tatABC *operon for *V. cholerae*. The *tatA2 *gene on chromosome II has a high degree of homology to both *E. coli *genes *tatA *(36.7%) and *tatE *(38.2%) (Fig. 1). Due to the higher level of sequence identity of the *V. cholerae tatA2 to E. coli tatE *than to *E. coli tatA *(Fig. 1), and due to its distant location from *tatABC*, *tatA2 *appears to be most similar to the *E. coli tatE *gene. Therefore, we renamed *tatA2 *as *V. cholerae tatE*.

**Figure 2:**
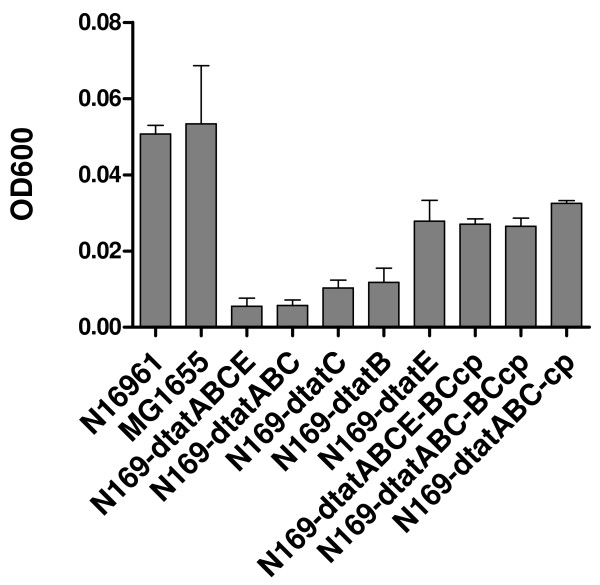
**Growth of *V. cholerae tat *mutants and complement strains in M9-TMAO media**. The OD_600 _was measured when the strains were cultured at 37°C for 24 h. The OD_600 _value for each strain was the average of three samples.

To determine whether the Tat mutants still have a functional Tat system, a series of Tat gene mutants of the *V. cholerae *strain N16961 was constructed to determine their growth in the M9-TMAO media. By using reverse transcription-PCR assay, transcription of corresponding *tat *genes in all the mutants and complement mutants were confirmed, each of the deleted genes were negative in reverse transcription-PCR, and all the complemented genes became positive in each complement strain (data not shown). In *E. coli*, Tat mutants were unable to grow anaerobically with either dimethyl sulfoxide or TMAO as the sole terminal electron acceptor, unless complemented by functional *tat *genes, due to the negligible levels of periplasmic TMAO reductase [32,33]. The *V. cholerae *mutants included deletion mutants of *tatABC *(N169-dtatABC), *tatABCE *(N169-dtatABCE), *tatB *(N169-dtatB), *tatC *(N169-dtatC) and *tatE *(N169-dtatE) (Table 1). The mutant *tatA *(N169-dtatABC-BCcp) was obtained by complementation with pBAD-TatBC into strain N169-dtatABC, and the double mutant strain (N169-dtatABCE-BCcp) of *tatA *and *tatE *was obtained by complementation with pBAD-TatBC into strain N169-dtatABCE (Table 1). We found that the wild type *V. cholerae *strain N16961 and MG1655, the *E. coli *strain derived from K-12, could grow in in M9-TMAO media, whereas the mutants N169-dtatABC and N169-dtatABCE could not grow after being cultured at 37°C for 24 h (Fig. 2). However, when pBAD-TatABC was restored into the mutants N169-dtatABC and pBAD-TatABC was restored into N169-dtatABCE, the complementary strains could grow well in the M9-TMAO media, indicating that the *tatABC *cluster is essential in the function of the Tat system. N169-dtatE and N169-dtatABC-BCcp could grow in M9-TMAO media, although the OD_600 _values of these strains were slightly lower than that of N16961 (Fig. 2). In addition, the OD_600 _of N169-dtatB and N169-dtatC was noticeably lower than that of N16961 in M9-TMAO media (Fig. 2). Therefore, the *tatB *and *tatC *genes appear to be necessary for the *V. cholerae *Tat system, and *tatA *and *tatE *may functionally overlap in *V. cholerae*.

**Figure 3:**
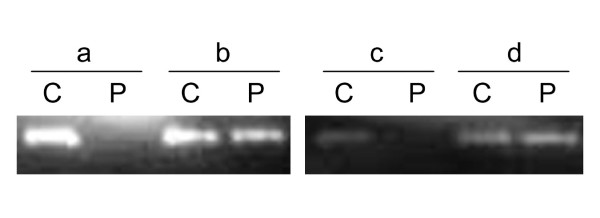
**TMAO reductase detection in both cytoplasm and periplasm by the TMAO enzymatic assay**. C, cytoplasm; P, periplasm; a, strain N169-dtatABC; b, strain N16961; c, strain N169-dtatABC (pBAD24); d, strain N169-dtatABC-cp.

We also transformed pBAD-TatABC and pBAD-TatE, plasmids containing *V. cholerae*-derived *tatABC *and *tatE*, into the *E. coli tat *gene mutants [34] to assess if TatA or TatE is essential to Tat system. As shown in Table 2, pBAD-TatABC restored the growth of *E. coli tatAE*, *tatB*, *tatC*, and *tatABCDE *mutants in M9-TMAO media, whereas pBAD-TatE only restored the growth of the *tatAE *mutant. Therefore, *V. cholerae tat *genes can replace their *E. coli *counterparts to reconstitute a heterologous functional Tat system. Here it was also shown that *tatE*, located on chromosome II, may functionally overlap *tatA *in *V. cholerae*. The functionality of the Tat system was also confirmed by the subcellular distribution of TMAO reductase activity in the wild type strain N16961, the *tatABC *mutant strain N169-dtatABC, and strain N169-dtatABC-cp, N169-dtatABC restored with pBAD-TatABC. The prepared fractions of periplasm and cytoplasm were confirmed with the control of western blot assay, using the antibodies to β-lactamase and GroEL. It was shown that β-lactamase was predominantly in the extractd periplasmic fraction, while GroEL was mainly in the extracted cytoplasmic fraction [see Additional file 2]. As anticipated, the TMAO reductase activity was detected in the periplasm of the wild type strain N16961 and N169-dtatABC-cp, but it accumulated in the cytoplasm of N169-dtatABC (Fig. 3).

**Table 2:** Using M9-TMAO media to detect the function of the Tat system in *E. coli *Tat mutant strains complemented with plasmids containing *V. cholerae tat *genes

Strains	pBAD24	pTatABC-301	pBAD-TatABC	pBAD-TatE
JARV16A (dtatAE)	-^a^	+	+	+
MCMTAA(dtatB)	-	+	+	-
B1LK0A (dtatC)	-	+	+	-
DADEA(dtatABCDE)	-	+	+	-

a: "-" or "+" means no-growth or successful growth of the strain in TMAO minimal media under anaerobic conditions, respectively.

### Growth and morphology of the *tatABC *mutant

**Figure 4:**
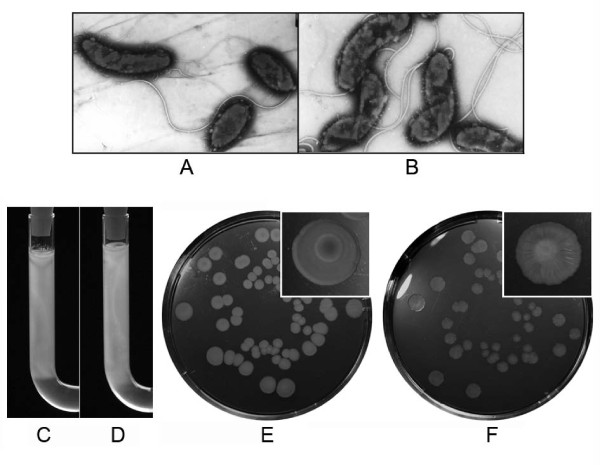
**Phenotypes of the *tatABC *mutant N169-dtatABC**. A, Electron micrograph of the wild type strain N16961 (×2400); B, Electron micrograph of the mutant N169-dtatABC (×2800); C, the motility of N169-dtatABC in 0.25% LBA, 37°C, 12 h; D, the motility of N16961 in 0.25% LBA, 37°C, 12 h; E and F, Smooth colonies of the wild type strain (E) and rugose colonies of the mutant N169-dtatABC (F) in LBA after 16 days in room temperature. The magnified inset images show the single colonies.

The *E. coli *Tat system is required for the translocation of amidases, and *tat *mutants display impaired cell division and chain-forming phenotypes [26]. We found that both the wild type strain and the *tatABC *mutant N169-dtatABC exhibited normal vibrioid morphology (Fig. 4A and 4B), except that some of mutant cells showed the curved or contorted form. The chains of bacterial cells of the mutant were not observed. Therefore, the Tat protein translocation system did not seem to obviously affect the cell morphology of N16961. Under both aerobic and anaerobic conditions at 37°C, the mutant strain N169-dtatABC did not show any obvious growth deficiencies (data not shown); hence, the Tat protein translocation system did not seem to affect its growth and division.

Like the wild type strain, the *tatABC *mutant colonies were smooth and moist in fresh LBA medium for the first 7 days at room temperature. Interestingly, in contrast to the wild type strain, some of N169-dtatABC colonies started to shift to the rugose (wrinkled) phenotype 7 days after inoculation at room temperature, and all the colonies of the mutant shifted to the rugose phenotype 16 days after inoculation, while colonies of the wild type strain were still smooth (Fig. 4E and 4F). Therefore, in contrast to the wild type strain, the *tatABC *mutant was easier to shift to the rugose phenotype at room temperature.

### Outer membrane integrity assay

To test the integrities of the outer membrane of *V. cholerae *tat mutants, we quantified the sensitivity of the mutants with respect to the hydrophobic drug Get and the detergent SDS, based on the concentration of Get or SDS that is required to kill 50% of the cells in liquid culture (LD50). LB without SDS or Get was used as the negative control. We compared the OD_600 _of the wild type strain and the mutant strains cultured in LB with different dilutions of SDS or Get, and did not find any changes of OD_600 _and LD50 when compared the wild type strain N16961 with the different *tat *gene mutants, therefore we did not find any integrity defect in the Tat mutants, including N169-dtatABCE, N169-dtatABC, N169-dtatB, N169-dtatC, and N169-dtatE (data not shown).

### Flagellum synthesis and motility

It has been reported that *tat *mutants lose motility and their flagellum synthesis is impaired [14]. We inspected the motility of *V. cholerae *N169-dtatABC in soft agar and found that the motility rate of the *tatABC *mutant was about 90% of that of the wild type strain (Fig. 4C and 4D), indicating that there is no significant influence of the *tat *mutation on the motility of cells.

To validate whether the *tatABC *mutation of *V. cholerae *impacts flagellum synthesis, flagella were extracted from N16961 and N169-dtatABC cells. The purity of the flagellum extracts in HEPES buffers was confirmed by denaturing SDS-PAGE (data not shown). The concentrations of the flagellum extracts from N16961 and N169-dtatABC cells were 1.328 μg/g and 1.303 μg/g of wet weight of bacterial culture, respectively. We did not find any difference in the amount of extracted flagellum protein between the N16961 and N169-dtatABC cells.

Flagella of the mutants were also observed under the electron microscope (Fig. 4B). Using fluorescence microscopy, we discovered that the motility of the Tat mutants was active. These results are consistent with the normal motility of the Tat mutant in minimal motility agar (Fig. 4C and 4D). Therefore, the Tat system of *V. cholerae *does not seem to influence flagellum synthesis or motility, unlike that of *E. coli *O157:H7 [14].

### Biofilm formation and CT production

**Figure 5:**
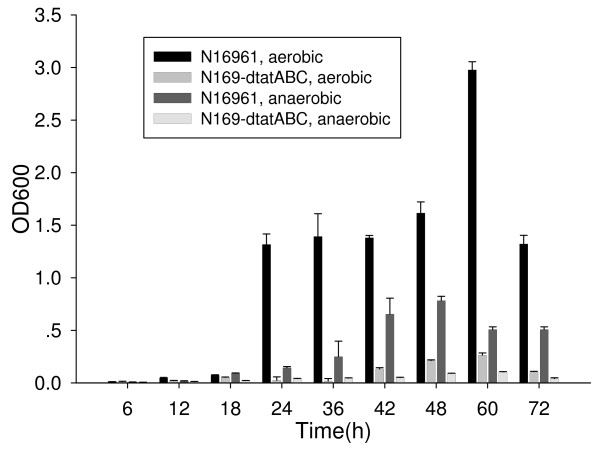
**Comparison of biofilm formation by strains N16961 and N169-dtatABC cultured under aerobic and anaerobic conditions**. For each strain (N16961 and n169-dtatABC), under each condition (aerobic and anaerobic), and at each time point, 7 wells were measured for repeat in one test. And the tests were repeated for three times. T-test was used for the comparison of strains N16961 and N169-dtatABC at each time point and under each condition. P values are less than 0.05 in all of the comparisons.

The ability to form biofilm formation is important for environmental survival and is a determining factor of virulence in pathogenic bacteria. To determine biofilm formation for the Tat mutants, we used a crystal violet staining method to quantify the adhering bacteria cultures in 96-well plates. Our findings indicate that under both aerobic and anaerobic conditions, the biofilm formation ability of the Tat mutant distinctly decreased (Fig. 5), which demonstrated that the Tat system of *V. cholerae *may play an important role in biofilm formation.

**Figure 6:**
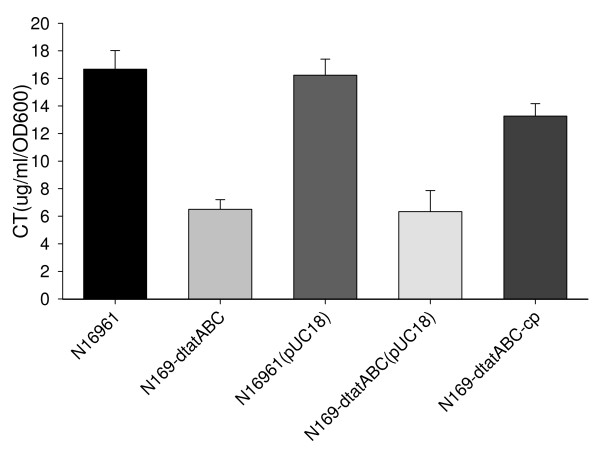
**CT production in the supernatant of strains N16961, N169-dtatABC, and N169-dtatABC-cp**. The strains were cultured using the AKI method. Data were obtained in independent triplicate cultures for each strain.

We also assessed cholera toxin (CT) production, which is secreted via the type II pathway [35-37]. To compare CT secretion of the wild type strain and *tat *mutants, we quantified CT production in the supernatant of N16961 and N169-dtatABC cells grown under AKI conditions by GM1-ELISA. Unexpectedly, the amount of CT secreted into the supernatant by the *tatABC *mutant strain was markedly less than that secreted by the wild type strain (7.3 μg/ml/OD_600 _for N169-dtatABC and 18.1 μg/ml/OD_600 _for N16961, P < 0.05 for the comparison of these two strains, One-Way ANOVA: Post Hoc Multiple Comparisons method, Fig. 6). To investigate if the decreased CT production was a consequence of the *tat *deletion, complementation experiments were also performed. When the mutant was complemented with pBAD24-tatABC, CT production of the N16961-dtatABC-cp strain increased compared to that of the mutant strains, N169-dtatABC and N169-dtatABC(pUC18) (P < 0.05 for the N16961-dtatABC-cp/N16961 comparison, and P < 0.05 for the N169-dtatABC-cp/N169-dtatABC comparison, One-Way ANOVA: Post Hoc Multiple Comparisons method, Fig. 6), indicating that the decrease in CT production in the supernatant of the mutant may result from a defect in the Tat system.

We also measured the amount of CT in the cytoplasm. The CT concentration in the cytoplasm of both N16961 and N169-dtatABC cells was much lower (< 5 ng/ml/OD_600_) than that in the culture supernatant (14–19 μg/ml/OD_600_), indicating that most of the CT was exported. The percentages of toxin secreted in the wild type strain and the *tatABC *mutant were nearly identical (99.97% and 99.93%, respectively). Although CT was still exported in the mutant, its production was markedly decreased compared to that of the wild type strain.

We then examined CT gene transcription in the tat mutant and wild type strain with quantitative RT-PCR. We determined that, for the *ctxB *gene, the difference ΔΔCt of N169-dtatABC/N16961 was 1.523 with *thyA *as the internal reference and 1.506 with the 16S rDNA gene as the internal reference. Based on 2^-ΔΔCt ^method, the *ctxB *gene transcription level of N169-dtatABC was 0.348 times compared to N16961 when using *thyA *as reference, and 0.352 times when using 16s-rDNA gene as reference, showing that cholera toxin gene was downregulated in the Tat mutant when compared to the wild type strain.

### *In vivo *colonization and *in vitro *cell attachment experiments

Colonization in the host intestine is required for the pathogenicity of *V. cholerae*. To analyze the colonization ability of the *tat *mutant strain, a suckling mouse intestine model was used in competitive experiments. We found that the colonization ability of the mutant was less than that of the wild type strain, as the colonization competitive ratio of the wild type strain N16961 to the mutant strain N169-dtatABC was 84:1 (from 40 to 120).

**Figure 7:**
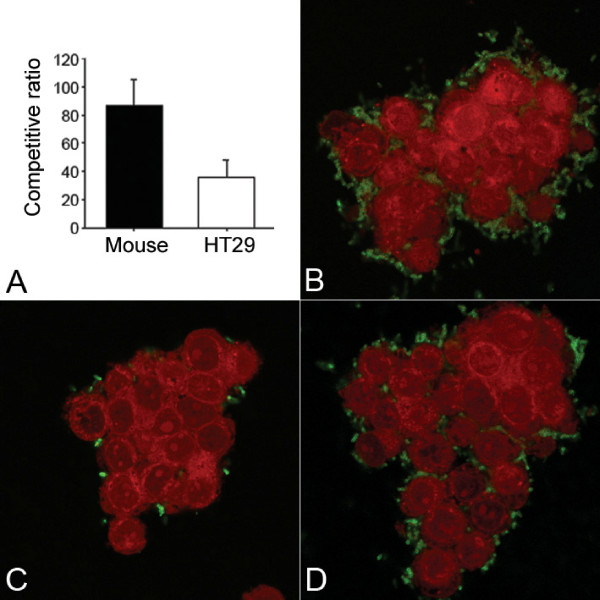
**Colonization and attachment attenuation of the *tatABC *mutant N169-dtatABC**. A. Colonization competitive ratio in suckling mouse model (left column, from 12 mice) and attachment competitive ratio for HT-29 cells (right column, from 6 repeats); B to D, Confocal imaging of the attachment of wild type strain N16961 (B), the *tatABC *mutant N169-dtatABC (C), and the complement strain N169-dtatABC-cp (D) to cultured HT-29 cells. The bacteria (green) were immunostained with FITC-labeled antibodies as described in Materials and Methods. HT-29 cells (red) were identified by Evan's blue staining.

Additionally, in the cell culture model, attachment to HT-29 was lower for the mutant than for the wild type strain (Fig. 7A to 7D). The attachment competitive ratio for the wild type strain N16961 to the mutant strain N169-dtatABC was 39: 1 (from 16 to 49). When the mutant strain was complemented with pTatABC-N16961, the attachment ability was restored (Fig. 7D).

## Discussion

In this study, we determined the functionality of the *tatABC *and *tatE *genes in *V. cholerae*. Our study demonstrates that the Tat functions are associated not only with the virulence of *V. cholerae *but also with its environmental survival. We found that the Tat system is functionally associated with biofilm formation and colonization ability in *V. cholerae*, and it may indirectly affect the production of cholera toxin.

In *E. coli*, *tatABC *forms an operon and *tatE *forms an independent transcriptional unit positioned away from *tatABC *[4]. Correspondingly, in *V. cholerae *strain N16961, *tatABC *is located in chromosome I, and *tatE *is located in chromosome II. By searching the GenBank we found the O1 classical biotype strain O395 also possesses *tatABC *and *tatE *homologous sequences, we speculate that the toxigenic serogroup O139 strains should also have the *tat *gene homologue. Whereas further study is needed to confirm the chromosomal distribution of the genes and functions. It is unclear why *V. cholerae *possesses two chromosomes, perhaps chromosome II plays a specialized independent role under evolutionary selective pressure [19]. It has been observed that several of the regulatory pathways, for regulation in response to both environmental and pathogenic signals, are divided between the two chromosomes. Also, duplications of genes with at least one of copy of the ORF were found on each chromosome. Most of these genes are involved in *V. cholerae *biology, notably its ability to inhabit diverse environments [19]. Therefore, the function of *tatE *in particular should be considered. By using reverse transcription PCR, we found that *tatE *in chromosome II is also transcribed independently (data not shown). It may not be a simple duplication of *tatA *in chromosome I because individual deficiency of *tatA *or *tatE *still impaired the anaerobic growth of mutants in M9-TMAO media in comparison to the wild type strain.

Biofilm formation is crucial for the survival of *V. cholerae *under environmental stress. The formation of biofilm can also make *V. cholerae *more resistant to acidic environments and increase its ability to break through the gastric acid barrier in humans [38]. In this study, we noticed that biofilm formation in the *tatABC *mutant was impaired, but it could be restored by complementation with functional *tatABC *genes. In *P. aeruginosa *[11] and *E. coli *[39], biofilm formation of the *tatC *mutants is also defective. It has been shown that the failure to form biofilms in the *E. coli tatC *mutant strain is due to defects in the cell envelope [39]. Some studies indicate that *tat *mutant strains display a pleiotropic lesion in their outer membrane [26,33]. Based on experiments on the sensitivity of the mutants to the hydrophobic drug Gentamicin and the detergent SDS, we did not find the defects in outer membrane integrity in the *V. cholerae tatABC *mutant. It is possible that Tat mutations may have pleiotropic effects in different bacteria, that the changed components in the membrane were not detected by our experiments, or that the changed components do not affect the membrane integrity.

Considering that the colonies of the *tatABC *mutant can shift to rugose type on LBA after extended time periods, some factors associated with biofilm formation and/or some membrane components are affected in the *tat *mutant. In comparison with the wild type strain, approximately 50% of the differentially expressed genes of the *E. coli tatC *mutant are linked to the envelope defect. Many of these genes are involved in self-defense or protection mechanisms, including the production of exopolysaccharides [39].

We found that the *V*. *cholerae tatABC *mutant can shift to the rugose phenotype and present "wrinkled" rather than typical smooth colonies on LB agar. In *E. coli, tatC *mutants routinely appear highly mucoid in comparison with the wild type strain when incubated on solid medium for extended periods of time. This result is thought to be due to the upregulation of some genes related to cell capsule formation in response to the cell envelope defect [39]. Rugose variants secrete copious amounts of exopolysaccharide, which confers resistance to chlorine, acidic pH, serum killing, and osmotic and oxidative stresses. Although the biofilm formation ability of N169-dtatABC decreased within the first three days in liquid culture, the rugose colony transformation capability of the mutant was enhanced when it was cultured at room temperature for longer times. When the rugose colonies of the mutant were transferred to fresh medium, the new colonies shifted exclusively to the smooth phenotype. We deduced that the *tatABC *mutant has a decreased ability to adapt to an environment with fewer nutrients in comparison with the wild type strain. Thus, the formation of rugose colonies of the Tat mutant might be a compensation response, which suggests that the Tat system may be involved in the environmental survival of *V. cholerae*.

Colonization in the host intestine is another important virulent factor for *V. cholerae*. We found that *tat *mutants displayed attenuated colonization competency in suckling mouse intestines and significantly attenuated attachment to HT-29 cells, even when slight differences in culture-growth curves under aerobic and anaerobic conditions were taken into consideration (within 10-fold). Based on these results, we believe that the Tat system may play a role the in maintenance of attachment and colonization in *V. cholerae*.

Several adherence factors have been described in *V. cholerae*, including outer membrane proteins (i.e., OmpU), hemagglutinins (i.e., mannose-sensitive hemagglutinin, MSHA), pili, and exopolysaccharides. Exopolysaccharides, MSHA and other factors have been proven to affect biofilm formation [40-43]. We speculate that some common factors responsible for adherence and biofilm formation might be affected in the *tat *mutant of *V. cholerae*, while the direct association might not exist.

Aside from biofilm formation and colonization, cholera toxin is the key virulence factor in the pathogenicity of *V. cholerae*. The activity of this enterotoxin primarily accounts for the clinical manifestations of *V. cholerae *infection. The mature secreted CT is composed of one A-subunit and 5 B-subunits. After translocation through the cytoplasmic membrane via the Sec pathway, the individual toxin subunits assemble noncovalently into an AB_5 _holotoxin complex in the periplasm and are then secreted across the outer membrane via the extracellular protein secretion apparatus [35-37]. In our study, we found that the cholera toxin output of the *tatABC *mutant strain was less than that of the wild type strain, but the ratio of CT secretion from the cytoplasm into the culture supernatant was the same.

Analysis of *ctxB *gene transcription revealed a lower level of transcription in the mutant than in the wild type strain. Therefore, the decrease in the amount of CT in the *tatABC *mutant may be due to lower production of CT in the mutant. This mechanism appears to differ from the effect of decreased secretion of the Shiga toxin 1 (Stx1) in the *tatC *mutant of *E. coli *O157:H7, which indicates that Tat may play an important role in secretion or stability of Stx1 [14]. Considering that the adherence and biofilm formation are also affected in the *tatABC *mutant of *V. cholerae*, further study is necessary to determine whether some global regulators responsible for these regulation pathways, their stability in the cytoplasm, or their anchoring in the membrane were affected.

The *tat *mutants of *E. coli *O157:H7 [14] and *A. tumefaciens *[13] lose their mobility, which is correlated with a defect in flagellum biogenesis. A dramatic effect on bacterial motility was also observed in the *tat *mutant of *P. aeruginosa*. It was presumed that the less motile phenotype was either an indirect effect of abnormal function of the flagella and pili, or the consequence of improper chemotaxis, or both [11]. In our experiments, an effect of flagellum biosynthesis by the *tatABC *mutation in *V. cholerae *was not found, and only slightly impaired motility was observed in the U tube tests. These observations illustrate that the effects of Tat may vary in different bacteria. For instance, the *tat *mutation obviously impairs cell growth rate in normal cultures of *A. tumefaciens *[13], *Mycobacterium smegmatis *[44], *P. aeruginosa *[11], and *E. coli *[33], whereas it was not affected in the mutants of *Y. pseudotuberculosis *[15] and *L. pneumophila *[17]. We also did not find a growth difference in LB culture between the *tat *mutant and the wild strain of *V. cholerae*.

## Conclusion

Our study demonstrates that the Tat system is functionally associated with biofilm formation and colonization ability in *V. cholerae*. In addition, it may indirectly affect the production of the cholera toxin, albeit not through a direct effect on its secretion. Seasonal cholera outbreaks in epidemic areas are linked to the persistence of *V. cholerae *in aquatic ecosystems, providing a reservoir for the initiation of new cholera epidemics via human ingestion of contaminated food or water, once the pathogens have traversed the gastric acid barrier of the stomach and colonized the intestine [45]. The requirement of the Tat system for the maintenance of biofilm formation may play an important role in *V. cholerae*'s persistence in aquatic ecosystems. Combined with the findings that a dysfunction in the Tat system can lead to attenuated virulence in other bacteria, Tat can be considered as an important virulence determinant of micropathogens. Therefore, the Tat functions are associated not only with the virulence of *V. cholerae *but also with its environmental survival. Gaining insight into their functionality is an important step in our understanding of the cholera and ultimately in the development of new therapies.

## Authors' contributions

LZ and ZZ performed most of the experiments in this study. LZ confirmed the function of *tatABC *in *V. cholerae*. ZZ constructed some new deletion mutants, repeated and complemented the data of the experiments, and prepared the draft. HJ provided plasmids, performed TMAO experiments, and conceived the experiments. JZ performed reverse transcription-PCR and confocal microscopy. YX performed the complementation assay of the *E. coli tat *gene mutants with the *tat *genes of *V. cholerae*. MY taught molecular techniques, performed cell culture, and provided critical discussion about the methodology. SG and JX participated in the design and coordination of the study. LF participated in the design and discussion of the study, and also provided *E. coli tat *mutants. BK designed and coordinated the study, and drafted the manuscript. All the authors read and approved the final manuscript.

## Authors' information

ZZ now is working in the Research Center of Shanghai Public Health Clinical Center Affiliated to Fudan University.

## Supplementary Material

Additional file 1 **Primers used to construct the recombinant plasmids and mutants of *tat *genes**. In this table the primer sequences used to construct recombinant plasmids, which were applied in construction of the mutants of *tat *genes, were listed. Click here for file

Additional file 2 **Localization of β-lactamase and GroEL in the fractions of *V. cholerae *strain N16961**. The image shows the activity of β-lactamase and GroEL detected in the fractions of *V. cholerae *strain N16961, to confirm the periplasmic and cytoplasmic fractions extracted from the whole cells of N16961. The proteins in the fraction of periplasm and cytoplasm were separated by SDS-PAGE and immunoblotted using mouse antibodies to β-lactamase and GroEL. The sizes of the marker were marked on the left. P: periplasmic fraction. C: cytoplasmic fraction. Click here for file
