# Supplementary material for: Pleiotropic effects of the twin-arginine translocation system on biofilm formation, colonization, and virulence in Vibrio cholerae
Source: BMC Microbiol. 2009 May 31;9:114. doi: 10.1186/1471-2180-9-114 (PMC2698830; doi:10.1186/1471-2180-9-114)
Supplement: Additional file 1 — Primers used to construct the recombinant plasmids and mutants of tat genes. In this table the primer sequences used to construct recombinant plasmids, which were applied in construction of the mutants of tat genes, were listed. [file 1471-2180-9-114-S1.doc]

**Additional file 1**

Table. Primers used to construct the recombinant plasmids and mutants of *tat* genes.

| **Fragments** | **Primers** | **Generated plasmids** |
| --- | --- | --- |
| To construct N169-dtatABC (“A-cat-B” type #) | | |
| A | cggaattcgagctcACTGGATACCAATGTTGATG (*EcoR*I *)  ggggtaccGATAAGAAGTTGCCAAATACTG (KpnI) | pT1 |
| B | gctctagaAAGCGTCCATACATTATC (XbaI)  aactgcagGTGTTAC GGCTCCTGATG (PstI) | pT2 |
| cat | CGTAGCACCAGGCGTTTAAG  GATCGGCACGTAAGAGGTTC | pT3 |
| To construct N169-dtatB (“A-B” type) | | |
| A | ggaattccACC AAG TGG GGC CAC AAG CTT TC (EcoRI)  cgagctcgACC AGT TCC CAA AAA CCG ATA TC (SacI) | pUC18-A |
| B | cgggatcccg GGA AAA AAA GGC CGA ATA AGC G (BamHI)  gctctagagc TAC AAA CGC ACC AAC GAT AAT G (XbaI) | pUC18-AB |
|  | aactgcag ACC AAG TGG GGC CAC AAG CTT TC PstI ??  acatgcatgc TAC AAA CGC ACC AAC GAT AAT G SphI ?? | Amplified from pUC18-AB, then cloned into pDS132 |
| To construct N169-dtatC (“A-B” type) | | |
| A | ggaattccAAA CAG CGC AGC AAA TCA GAA AG  cgagctcgTGT TCA ACG GAA GAC ATG CAA AC | pUC18-A |
| B | cgggatcccg GGA CAA GAG GAA GAA GAA TAA AG  gctctagagcGCG CAG GTA AAA TGG GGG AAA C | pUC18-AB |
|  | aactgcag AAA CAG CGC AGC AAA TCA GAA AG  acatgcatgc GCG CAG GTA AAA TGG GGG AAA C | Amplified from pUC18-AB, then cloned into pDS132 |
| To construct N169-dtatE (“A-B” type) | | |
| A | ggaattccAAG ACG GCA TCA GTT TGA CGA G  ggggtaccccGCT TAC CCA CAC TGA TTC CAC C (KpnI) | pUC18-A |
| B | cgggatcccg CAG CGC CAC TCA TAA TGT TCC C  gctctagagc CTA TTC CTC GCG GGC TGG TTG G | pUC18-AB |
|  | cgagctcg AAG ACG GCA TCA GTT TGA CGA G  acatgcatgc CTA TTC CTC GCG GGC TGG TTG G | Amplified from pUC18-AB, then cloned into pDS132 |
| To construct the complementary plasmids | | |
| tatABC | aac tgc ag ATG CGG CTT TGT TTA ATC ATC  aca tgc atg c TTA TTC TTC AGT TTT TTC GCT | pTatABC-301  (*E. coli*) |
| tatABC | ggaattcc  GTG TTA TGT TGG CTA TTG AGT  gctctagagc CAG CGC TCA AAG CAC GGC TCT | pBAD-TatABC  (*V. cholerae*) |
| tatBC | ggaattcc GTG TTT GAT ATC GGT TTT TGG  gctctagagc TTA TTC TTC TTC CTC TTG TCC |  |
| tatE | ggaattcc GAT AAA TAG GCA CGT AAA ATA  cgagctcg TTA TGA GTG GCG CTG AGG ATC |  |

#: Amplified fragment A or B, please see the part of “Construction of the tat deletion mutants of *V. cholerae* N16961 by allelic replacement” in “Methods”.

* : Restriction site introduced into the primer (showed in small letters).
