# Supplementary figures and images for: Pleiotropic effects of the twin-arginine translocation system on biofilm formation, colonization, and virulence in Vibrio cholerae
Source: BMC Microbiol. 2009 May 31;9:114. doi: 10.1186/1471-2180-9-114 (PMC2698830; doi:10.1186/1471-2180-9-114)

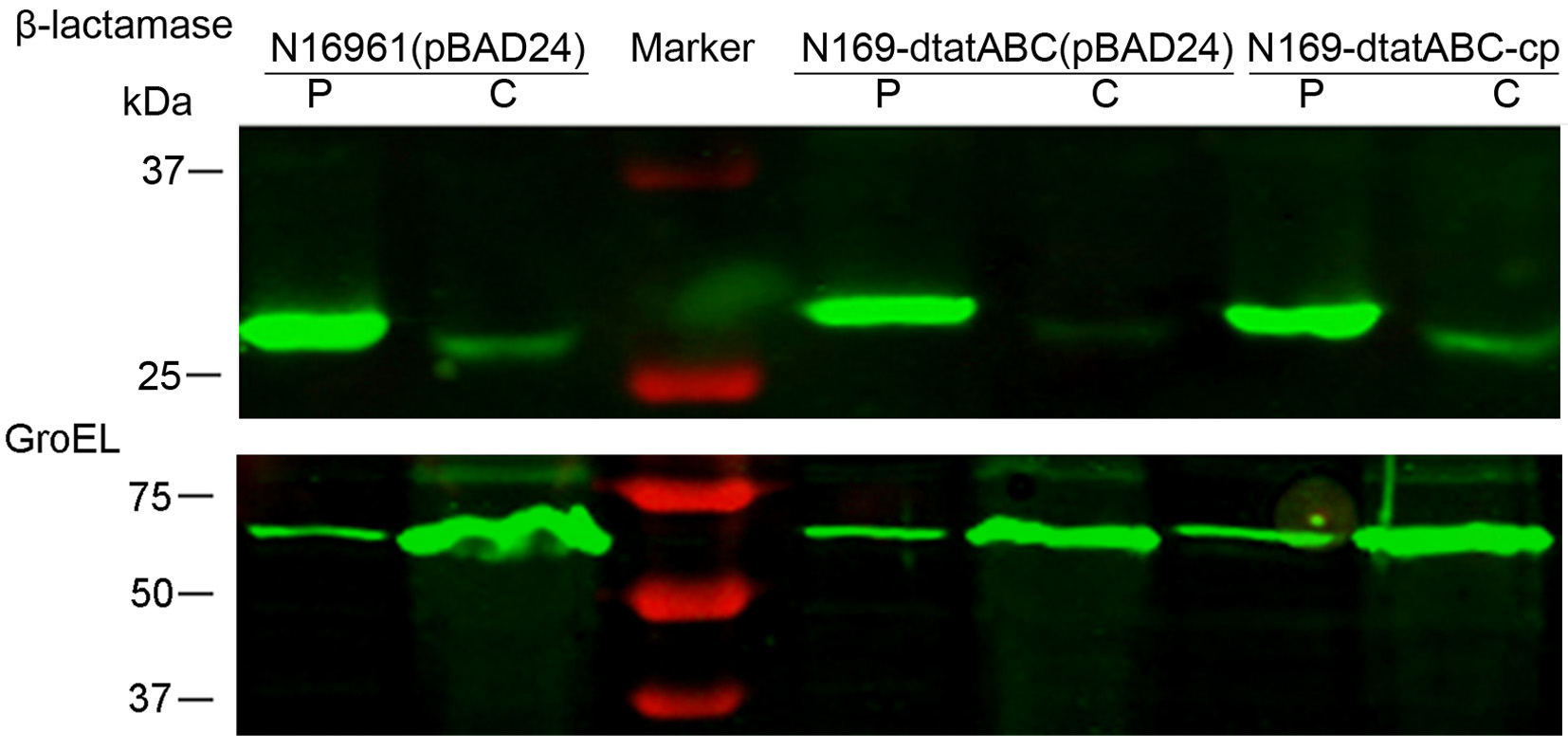

Supplement: Additional file 2 — Localization of β-lactamase and GroEL in the fractions of V. cholerae strain N16961. The image shows the activity of β-lactamase and GroEL detected in the fractions of V. cholerae strain N16961, to confirm the periplasmic and cytoplasmic fractions extracted from the whole cells of N16961. The proteins in the fraction of periplasm and cytoplasm were separated by SDS-PAGE and immunoblotted using mouse antibodies to β-lactamase and GroEL. The sizes of the marker were marked on the left. P: periplasmic fraction. C: cytoplasmic fraction. [file 1471-2180-9-114-S2.jpeg]
